# A novel violet/blue light-emitting device based on Ce_2_Si_2_O_7_

**DOI:** 10.1038/srep16659

**Published:** 2015-11-13

**Authors:** Ling Li, Shenwei Wang, Guangyao Mu, Xue Yin, Kai Ou, Lixin Yi

**Affiliations:** 1 Key Laboratory of Luminescence and Optical Information, Ministry of Education, Institute of Optoelectronic Technology, Beijing Jiaotong University, Beijing, 100044, China

## Abstract

Rare-earth silicates are highly efficient materials for silicon-based light sources. Here we report a novel light-emitting device based on Ce_2_Si_2_O_7_. Intense violet/blue electroluminescence was observed, with a turn-on voltage of about 13 V. The violet/blue emission is attributed to 4f–5d transitions of the Ce^3+^ ions in Ce_2_Si_2_O_7_, which are formed by interfacial reaction of CeO_2_ and Si. Electroluminescence and photoluminescence mechanisms of the Ce_2_Si_2_O_7_ light-emitting device are also discussed.

Silicon-based light-emitting devices have attracted considerable attention, since silicon has many advantages such as low fabrication cost, mature fabrication and processing technology, and high electrical and thermal conductivities^1^. In the past decades, integrations of ZnO, GaN, and other III-V semiconductors with silicon were extensively studied for novel optoelectronic applications because of the direct and wide band gaps of these materrials^2^,^3^,^4^. However, the lattice and thermal mismatch between these materials and Si still presents a significant challenge^5^,^6^. Alternatively, rare-earth doped SiO_2_ has attracted a lot of interest due to their high luminescence efficiency and wide spectral range spanning from ultraviolet (UV) to infrared (IR). More importantly, SiO_2_ is naturally compatible with Si technology^7^,^8^. Previously, efficient visible light-emitting devices based on rare-earth doped metal-oxide-semiconductor (MOS) structures have been demonstrated^9^,^10^,^11^. However, the solubility of rare-earth ions in Si-based materials achieved so far is still relatively low (10^16^−10^20^ cm^−3^)^12^. In fact, even if the solubility could be improved, since the rare-earth ions (e.g. Er^3+^, Eu^3+^, Ce^3+^) are optically inactive at high concentrations due to the formation of clusters^13^,^14^,^15^, such a concentration quenching would still be a significant barrier for further improving performance of rare-earth doped Si-based materials. Recently, it was shown that formation of rare-earth silicates (e.g. Er_2_SiO_5_, Eu_2_SiO_5_, Ce_2_Si_2_O_7_) could enhance their photoluminescence efficiency^16^,^17^,^18^. It was found that in these silicates, the rare-earth ions are 100% activated, and the solubility can reach 10^22^ cm^−3 ^19,20,21.

Ce_2_Si_2_O_7_ is a promising material for UV optoelectronic devices, with an emission band similar to that of ZnO and GaN. In addition, different emission wavelengths could be obtained from the energy transfer between Ce^3+^ and other rare-earth ions (such as Er^3+^, Tb^3+^, Dy^3+^, Sm^3+^)^10^,^11^,^22^,^23^. Although photoluminescence (PL) of Ce_2_Si_2_O_7_ has been investigated by several groups^16^,^18^,^24^,^25^, there has been no reports on electroluminescence (EL) of Ce_2_Si_2_O_7_ so far. In this paper, Ce_2_Si_2_O_7_ is synthesized by interfacial reaction of CeO_2_ thin film and Si wafer in reducing ambient. Violet/blue emissions at about 390 and 410 nm from Ce_2_Si_2_O_7_ were obtained. The internal quantum efficiency of Ce_2_Si_2_O_7_ was found to be about 37% at room temperature. Finally, we fabricated Ce_2_Si_2_O_7_ light-emitting devices (LEDs), and achieved a turn on voltage of 13 V, while the maximum EL intensity was obtained at a driving voltage of 25 V.

## Results and Discussion

**Figure 1:**
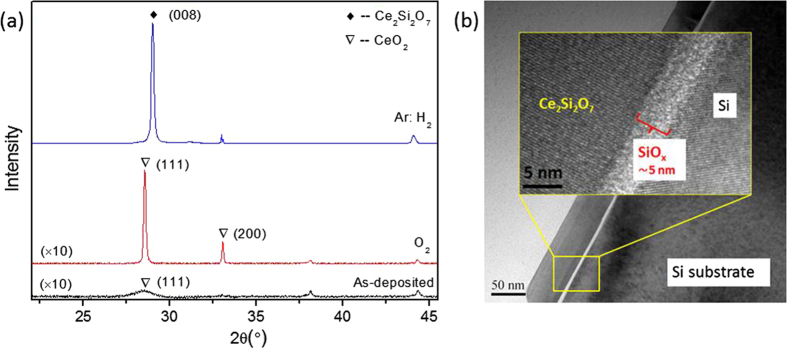
(a) XRD of as-deposited and annealed CeO_2_ films at 1060 °C for 1 hour, (b) TEM image of the Ce_2_Si_2_O_7_ film. The inset shows high-magnification TEM of the interface region marked by the yellow box.

In order to investigate the effect of annealing ambient on the structural change of CeO_2_ films, X-ray diffraction (XRD) patterns of as-deposited and annealed CeO_2_ films were measured. As shown in Fig. 1(a), the crystal structure of the samples depends strongly on the annealing ambient. The as-deposited film contains a broad reflection around 28.6°, which corresponds to the (111) plane of CeO_2_. When the as-deposited film was annealed in O_2_ ambient, the diffraction peak at 28.6° becomes higher and narrower, and the (200) plane of CeO_2_ can be identified. The Ce_2_Si_2_O_7_ phase is obtained after the as-deposited CeO_2_ film was annealed in reducing ambient, and the highest reflection peak is identified as the (008) plane. The lattice structure is also investigated by transmission electron microscopy (TEM). As shown in Fig. 1(b), the thickness of Ce_2_Si_2_O_7_ is about 50 nm. There is an amorphous SiO_x_ layer at the Ce_2_Si_2_O_7_/Si interface, with a thickness of about 5 nm. During the annealing process, CeO_2_ was reduced to CeO_2−x_, while Si was oxidized. Then, SiO_x_ and CeO_2−x_ reacted at high temperature, resulting in Ce_2_Si_2_O_7_ with various crystallographic orientations.

**Figure 2:**
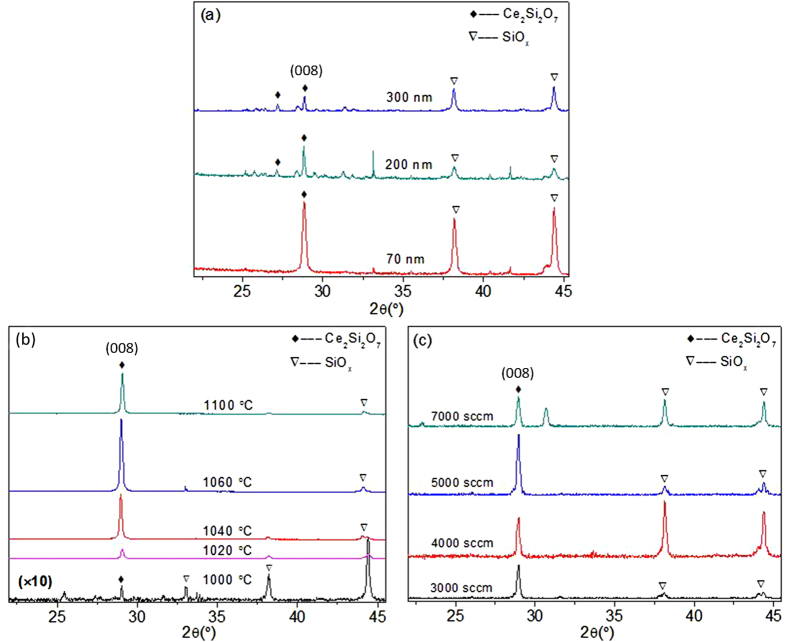
(**a**) XRD of as-deposited CeO_2_ films with different thicknesses annealed at 1020 °C for 1 hour, (**b**) XRD of 70 nm CeO_2_ films annealed at various temperatures for 1 hour, (**c**) XRD of 70 nm CeO_2_ films annealed at 1040 °C by different annealing ambient flow rate.

The lattice structure of Ce_2_Si_2_O_7_ observed is different from previous reports^18^,^26^,^27^. We attribute this to the strong dependence of the crystallinity and orientation on deposition and annealing conditions^27^,^28^,^29^. To confirm this, the as-deposited CeO_2_ films with different thicknesses were annealed in reducing ambient (Ar:H_2_ = 97:3) for 1 hour. As shown in Fig. 2(a), there are various crystallographic orientations of Ce_2_Si_2_O_7_ when 300 nm CeO_2_ film was annealed at 1020 °C. However, the intensity of the (008) diffraction peak increases with decreasing the thickness of CeO_2_. In Fig. 2(b), various crystallographic orientations of Ce_2_Si_2_O_7_ are exhibited when a 70 nm CeO_2_ film was annealed at 1000 °C. Unlike the 300 nm film, here the (008) diffraction peak initially increases with the annealing temperature up to 1020 °C, and then decreases while the annealing temperature is further raised to above 1060 °C. Similarly, as shown in Fig. 2(c), this peak first increases with the annealing ambient flow rate is increased, and then decreases once the flow rate up is above 5000 sccm. In summary, these observations show that the deposition and annealing conditions are critical factors for synthesizing Ce_2_Si_2_O_7_.

**Figure 3:**
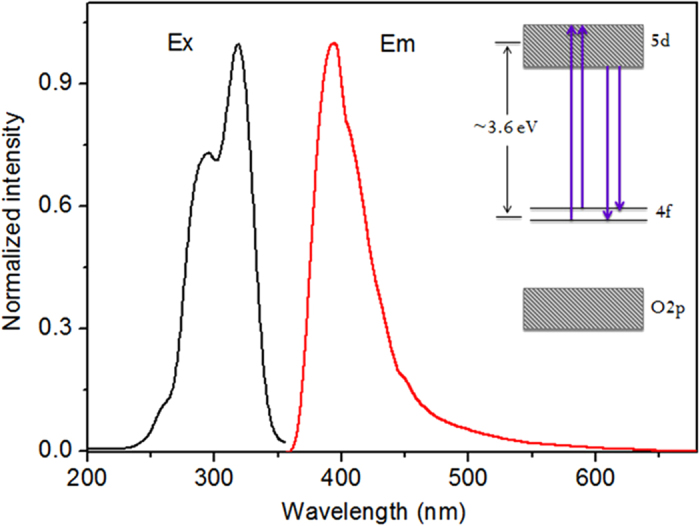
PL and PLE spectra of the CeO_2_ film annealed in Ar:H_2_ ambient with the excitation wavelength of 325 nm, the PLE was measured with a detection wavelength of 390 nm. The inset shows the energy levels diagram of Ce_2_Si_2_O_7_. All the spectra were measured at room temperature.

Figure 3 shows the PL spectrum of the CeO_2_ film annealed in Ar:H_2_ ambient. The intense violet/blue emission consists of two peaks centered at about 390 nm (3.18 eV) and 410 nm (3.02 eV), respectively. Both the shape and the position of the peaks are similar to the results obtained by Choi^18^. However, these emission bands are not observed in the as-deposited films or the samples annealed in O_2_ ambient. Combined with crystal structure analysis mentioned above, the violet/blue emission bands are attributed to the formation of Ce_2_Si_2_O_7_, and correspond to the Ce^3+^ transitions from the relaxed lowest 5d excited state to 4f ground states. The energy separation of the two peaks is about 2000 cm^−1^, which matches well with the theoretical value of spin–orbit splitting between the 4f ground state ^2^F_5/2_ and ^2^F_7/2_ 30. The excitation bands located at 295 nm (4.2 eV) and 324 nm (3.83 eV) can be assigned to the 4f–5d transitions^31^. Based on these assignments, a simplified energy level diagram is sketched in Fig. 3. In order to estimate the optical band gap (*E*_*g*_^*opt*^) of Ce_2_Si_2_O_7_, we measured the absorption spectrum of the sample in the visible range, and obtain a value of 3.6 eV by extrapolating the spectrum using Tauc’s relation^32^:


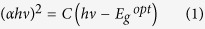


where *α* is the absorption coefficient, *h* is the Planck’s constant, *ν* is the photon frequency, and *C* is a constant.

**Figure 4:**
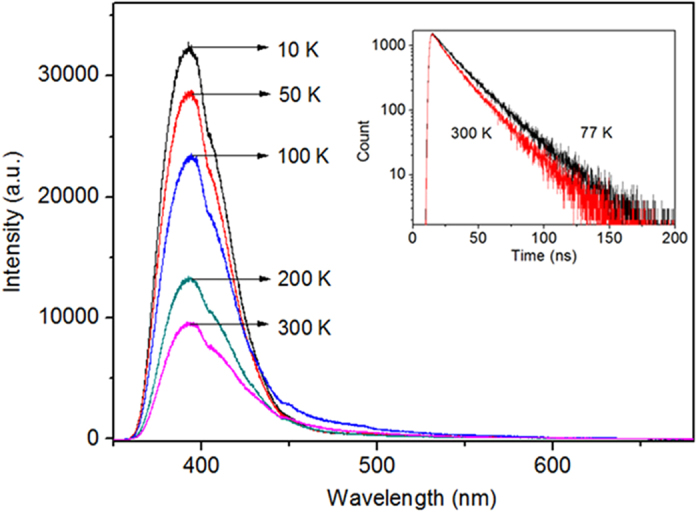
PL spectra from Ce_2_Si_2_O_7_ in the temperature range from 10 to 300 K with the excitation wavelength of 325 nm. The inset shows the PL decay of Ce_2_Si_2_O_7_.

According to several previous studies, Ce^3+^ activator concentration is limited to about 0.5 at% in most host materials^33^,^34^,^35^. However, our results show that Ce^3+^ ions in Ce_2_Si_2_O_7_ are almost 100% activated. This is similar to self-activated materials such as CeF_3_ and CeCl_3_ 36. We find that Ce^3+^ emission is enhanced significantly by the formation of Ce_2_Si_2_O_7._ To quantify this enhancement, we measured the internal quantum efficiency of Ce_2_Si_2_O_7_ by using an integrated sphere^37^. We obtained a value of about 37% at room temperature. To reveal the excitation and emission mechanisms, we measured the PL spectra as a function of temperature. As shown in Fig. 4, the PL intensity increases as the sample temperature is decreased. We also measured PL decay time at temperatures of 77 and 300 K, respectively, as shown in the inset of Fig. 4. The PL decays exponentially, with the time constant slightly changes from 17 to 21 ns as the temperature is decreased from 300 to 77 K. This very short lifetime is in agreement with the electric-dipole allowed 4f–5d transition rate of Ce^3+^. Since non-radiative centers are frozen at low temperatures, we attribute the decrease of the PL intensity and the decay time to the energy transfer to defect centers by non-radiative recombination processes^38^.

**Figure 5:**
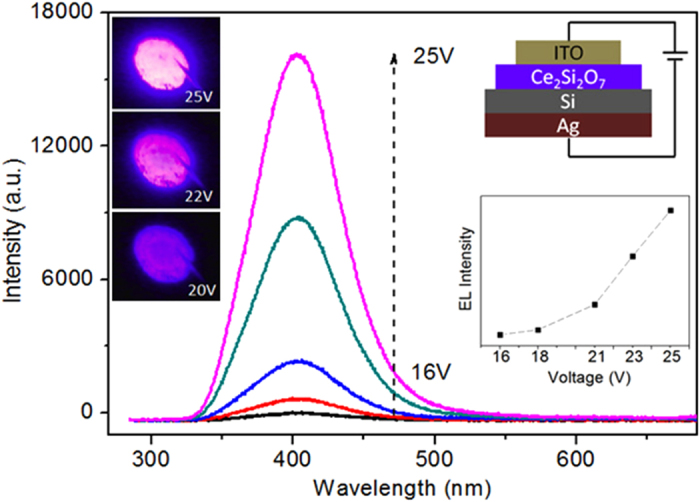
EL spectra of the LED at various forward biases of 16–25 V. The insets show the structure diagram of the LED and EL photos of the device at different voltages.

**Figure 6:**
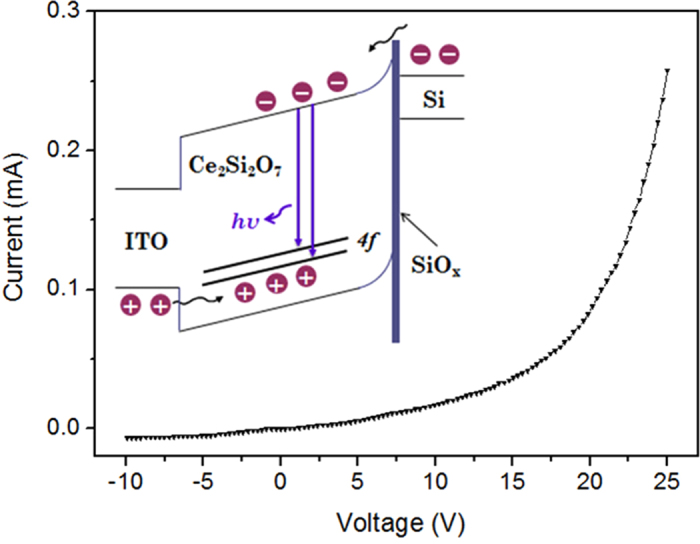
Current-voltage characteristic of the device. The inset shows the energy band diagram of the devices and the charge transfer process.

Next, we fabricated Ce_2_Si_2_O_7_ LEDs with the structure diagram shown in Fig. 5. Violet/blue EL emission is clearly observed when a positive voltage is applied on the indium tin oxide (ITO) layer. The turn-on voltage of the device is found to be as low as 13 V. The EL spectra (Fig. 5) are in good agreement with the PL spectra (Fig. 3). The EL intensity increases with the forward voltage, and the maximum EL intensity is obtained at 25 V. Figure 6 presents a typical current-voltage (I-V) characteristics. The forward current of the device reaches up to 0.26 mA when the forward bias is 25 V, while a reverse leakage current of 6.4 μA at a bias voltage of −10 V is observed. These values illustrate the excellent rectification performance achieved in this device.

Based on the energy level diagram of Ce_2_Si_2_O_7_ developed in this work, we propose the following mechanism of the LED operation: As seen from the inset of Fig. 6, an asymmetrical energy barrier is formed at the junction interface. When a positive voltage is applied between the two electrodes (forward bias), the electrons accumulated in the Si/SiO_x_ interface are swept to Ce_2_Si_2_O_7_ side by tunneling through the SiO_x_ barrier. Meanwhile, the holes are injected into Ce_2_Si_2_O_7_ from ITO and accumulated at Ce_2_Si_2_O_7_/SiO_x_ interface due to the SiO_x_ barrier. As a result, the EL originates from the recombination between the injected electrons and holes in Ce_2_Si_2_O_7_. These emitted photons with an energy (hυ) approximately equals to the energy difference between 4f and 5d states of Ce^3+^. Hence, in this design, the SiO_x_ layer functions as a carrier blocking layer. For high-performance and reliable device operation, the ideal thickness of SiO_x_ layer should be less than 2 nm^39^. Therefore, the turn-on voltage can be further decreases, considering the 5 nm SiO_x_ layer used in this device.

In summary, a new method of synthesizing Ce_2_Si_2_O_7_ was demonstrated. Ce_2_Si_2_O_7_ was formed after the Si-based CeO_2_ film was annealed in reducing ambient. Intense violet/blue emission was observed from Ce_2_Si_2_O_7_, and the PL emission bands are located around 390 and 410 nm, which are attributed to 4f–5d transitions of Ce^3+^. More importantly, Ce_2_Si_2_O_7_ LEDs were fabricated and strong violet/blue EL emission was observed. The turn on voltage of Ce_2_Si_2_O_7_ LED is 13 V and the maximum EL intensity was obtained at 25 V.

## Methods

CeO_2_ thin films were deposited on P-type Si (100) substrates by electron beam evaporation (EVA 450). The as-deposited films were annealed in reducing ambient (Ar:H_2_ = 97:3) at various temperatures for 1 hour. We fabricated the LEDs as schematically illustrated in the inset of Fig. 5. ITO and Ag electrodes were deposited on the surface of the film and the back side of Si substrates, respectively, both by magnetron sputtering. The PL spectra were measured by a He-Cd laser with a 325 nm excitation wavelength, and the PL excitation (PLE) spectrum was measured with a fluorescence spectrometer (FLS920) using a 450  W xenon lamp as the excitation source. The crystal structures were characterized by XRD using Cu Kα radiation (Bruker D8 ADVANCE), and morphology of the samples was determined by TEM (Hitachi, H8100 200 kV). The internal quantum efficiency of samples is measured using an F-3018 integrating sphere with a 335 nm laser excitation. The PL decay was measured by using a nanosecond xenon flash lamp at 325 nm excitation wavelength and detected by a time-correlated single photon-counting system. The EL spectra of the devices and I-V characteristics were measured by a system of an ACTON 150 CCD spectrometer and a Keithley 2410, respectively.

## Additional Information

**How to cite this article**: Li, L. *et al.* A novel violet/blue light-emitting device based on Ce_2_Si_2_O_7_. *Sci. Rep.* **5**, 16659; doi: 10.1038/srep16659 (2015).
